# *catena*-Poly[[[bis­(thio­cyanato-κ*N*)zinc]bis­[μ-1,3,5-tris­(1*H*-1,2,4-triazol-1-yl­meth­yl)benzene-κ^2^ *N* ^4^:*N* ^4′^]] mono­hydrate]

**DOI:** 10.1107/S1600536812039256

**Published:** 2012-09-26

**Authors:** Qing-Xia Li, Xian-Ju Shi, Lai-Cheng Chen

**Affiliations:** a Department of Petroleum & Chemical Engineering, Puyang Vocational and Technical College, Puyang 457000, People’s Republic of China

## Abstract

In the title complex, {[Zn(NCS)_2_(C_15_H_15_N_9_)_2_]·H_2_O}_*n*_, the Zn^II^ ion is located on an inversion centre and is six-coordinated in a distorted octa­hedral geometry, coordinated by N atoms from four bridging 1,3,5-tris­(1,2,4-triazol-1-ylmeth­yl)benzene (ttmb) ligands and two terminal SCN^−^ counter-anions. Two of the three triazol groups in each ttmb ligand link the Zn^II^ atoms, forming a looped-chain structure along [0-11]. The lattice water molecule shows half-occupancy due to disorder around an inversion centre.

## Related literature

For background to the use of flexible tripodal compounds in the design and construction of compounds with metal-organic framework structures, see: Moon *et al.* (2006 ▶); Xu *et al.* (2009 ▶). For similar structures, see: Yin *et al.* (2009 ▶); Shi *et al.* (2011 ▶).
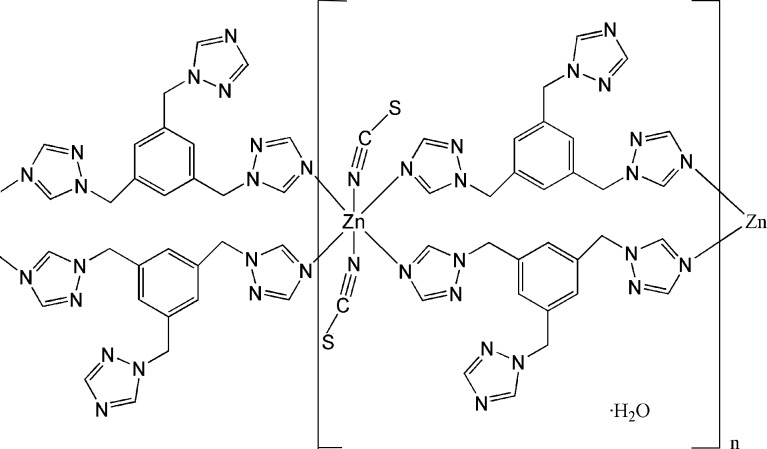


## Experimental

### 

#### Crystal data

[Zn(NCS)_2_(C_15_H_15_N_9_)_2_]·H_2_O
*M* *_r_* = 842.27
Triclinic, 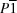

*a* = 8.5766 (17) Å
*b* = 9.5036 (19) Å
*c* = 11.723 (2) Å
α = 80.01 (3)°
β = 85.40 (3)°
γ = 89.55 (3)°
*V* = 938.0 (3) Å^3^
*Z* = 1
Mo *K*α radiation
μ = 0.83 mm^−1^
*T* = 293 K
0.20 × 0.18 × 0.16 mm

#### Data collection

Rigaku Saturn724 diffractometer
Absorption correction: multi-scan (*CrystalClear*; Rigaku/MSC, 2006 ▶) *T*_min_ = 0.852, *T*_max_ = 0.879
11566 measured reflections
4444 independent reflections
3972 reflections with *I* > 2σ(*I*)
*R*_int_ = 0.024

#### Refinement

*R*[*F* ^2^ > 2σ(*F* ^2^)] = 0.041
*wR*(*F* ^2^) = 0.108
*S* = 1.05
4444 reflections
265 parameters
9 restraints
H atoms treated by a mixture of independent and constrained refinement
Δρ_max_ = 0.61 e Å^−3^
Δρ_min_ = −0.39 e Å^−3^

### 

Data collection: *CrystalClear* (Rigaku/MSC, 2006 ▶); cell refinement: *CrystalClear* (Rigaku/MSC, 2006 ▶); data reduction: *CrystalClear* (Rigaku/MSC, 2006 ▶); program(s) used to solve structure: *SHELXS97* (Sheldrick, 2008 ▶); program(s) used to refine structure: *SHELXL97* (Sheldrick, 2008 ▶); molecular graphics: *CrystalStructure* (Rigaku/MSC, 2006 ▶); software used to prepare material for publication: *CrystalStructure* (Rigaku/MSC, 2006 ▶).

## Supplementary Material

Crystal structure: contains datablock(s) global, I. DOI: 10.1107/S1600536812039256/ds2211sup1.cif

Structure factors: contains datablock(s) I. DOI: 10.1107/S1600536812039256/ds2211Isup2.hkl

Additional supplementary materials:  crystallographic information; 3D view; checkCIF report

## Figures and Tables

**Fig. 1.**
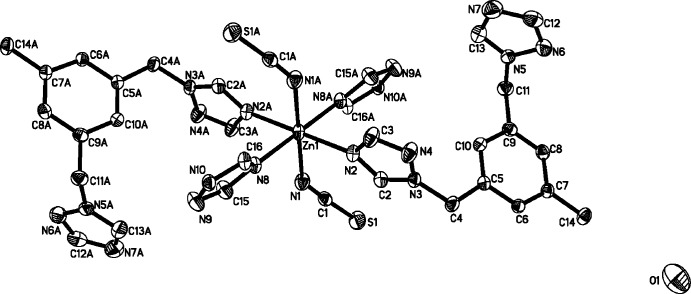
A fragment of the title complex, showing the coordination environment of ZnII center with atom labelling of the non-H atoms and 30% probability ellipsoids. H atoms have been omitted.

**Fig. 2.**
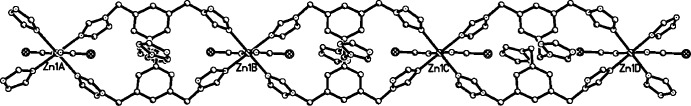
View of the one-dimensional looped-chain structure of the title complex.

## supplementary crystallographic information

### Comment

Flexible tripodal compounds are known to be the versatile structural constructors in the rational design and construction of novel metal-organic frameworks (MOFs), in respect that its three potential coordination groups can bend and rotate freely to satisfy various coordination preferences and facilitate the formation of various complexes with diverse structures and properties (Moon *et al.*, 2006; Xu *et al.*, 2009). Therefore, the prospect of exploring the influential principles of tripodal compounds on the resulting framework structures provides an impetus for further researches on tripodal compounds. We were thus engaged in the synthesis of a flexible tripodal N-heterocyclic compound 1,3,5-tris(1,2,4-triazol-1-ylmethyl)-benzene (ttmb) (Yin *et al.*, 2009; Shi *et al.*, 2011), and employed it as a ligand to construct a new complex {[Zn(SCN)_2_(ttmb)_2_].H_2_O}_n_. In the title complex, Zn^II^ ion is six-coordinated in a distorted octahedral geometry, coordinated by N2, N2A, N8 and N8A from four ttmb ligands, and N1, N1A from two terminal counter-anion SCN^-^ (Fig. 1). In ttmb, the center of one triazol ring lies inside the benzene plane with the dihedral angle of 89.4 °, and the other two triazol rings lie in the opposite orientation outside the plane to form an infrequent *trans* conformation. Two of the three triazol groups in each ttmb link the Zn^II^ centers together to form an one-dimensional looped-chain structure (Fig. 2), in which the Zn^II^ ions are collinear with the adjacent Zn···Zn distance of 13.8 Å.

### Experimental

A reaction mixture of ZnSO_4_.7H_2_O (29 mg, 0.1 mmol), 1,3,5-tris(1,2,4-triazol-1-ylmethyl)-benzene (ttmb) (32.1 mg, 0.1 mmol), KSCN (19.4 mg, 0.2 mmol), and 10 ml water was sealed in a Teflon-lined stainless steel vessel, which was heated at 130 °C for 72 h, and then cooled to room temperature, obtaining colorless crystals of the title complex. Yield (based on Zn): 31%.

### Refinement

H atoms were generated geometrically and refined as riding atoms with C-H = 0.93 Å, 0.97 (CH_2_) Å and Uiso(H) = 1.2 times Ueq(C).

### Crystal data

**Table:**

[Zn(NCS)_2_(C_15_H_15_N_9_)_2_]·H_2_O	*Z* = 1
*M**_r_* = 842.27	*F*(000) = 434
Triclinic, *P*1	*D*_x_ = 1.491 Mg m^−^^3^
*a* = 8.5766 (17) Å	Mo *K*α radiation, λ = 0.71073 Å
*b* = 9.5036 (19) Å	Cell parameters from 2773 reflections
*c* = 11.723 (2) Å	θ = 2.2–27.9°
α = 80.01 (3)°	µ = 0.83 mm^−^^1^
β = 85.40 (3)°	*T* = 293 K
γ = 89.55 (3)°	Prism, colorless
*V* = 938.0 (3) Å^3^	0.20 × 0.18 × 0.16 mm

### Data collection

**Table:**

Rigaku Saturn724 diffractometer	4444 independent reflections
Radiation source: fine-focus sealed tube	3972 reflections with *I* > 2σ(*I*)
Graphite monochromator	*R*_int_ = 0.024
Detector resolution: 28.5714 pixels mm^-1^	θ_max_ = 27.9°, θ_min_ = 2.2°
ω and φ scans	*h* = −11→11
Absorption correction: multi-scan ?	*k* = −12→12
*T*_min_ = 0.852, *T*_max_ = 0.879	*l* = −15→15
11566 measured reflections	

### Refinement

**Table:**

Refinement on *F*^2^	Primary atom site location: structure-invariant direct methods
Least-squares matrix: full	Secondary atom site location: difference Fourier map
*R*[*F*^2^ > 2σ(*F*^2^)] = 0.041	Hydrogen site location: inferred from neighbouring sites
*wR*(*F*^2^) = 0.108	H atoms treated by a mixture of independent and constrained refinement
*S* = 1.05	*w* = 1/[σ^2^(*F*_o_^2^) + (0.0517*P*)^2^ + 0.3475*P*] where *P* = (*F*_o_^2^ + 2*F*_c_^2^)/3
4444 reflections	(Δ/σ)_max_ < 0.001
265 parameters	Δρ_max_ = 0.61 e Å^−^^3^
9 restraints	Δρ_min_ = −0.39 e Å^−^^3^

### Special details

**Table:**

Geometry. All e.s.d.'s (except the e.s.d. in the dihedral angle between two l.s. planes) are estimated using the full covariance matrix. The cell e.s.d.'s are taken into account individually in the estimation of e.s.d.'s in distances, angles and torsion angles; correlations between e.s.d.'s in cell parameters are only used when they are defined by crystal symmetry. An approximate (isotropic) treatment of cell e.s.d.'s is used for estimating e.s.d.'s involving l.s. planes.
Refinement. Refinement of *F*^2^ against ALL reflections. The weighted *R*-factor *wR* and goodness of fit *S* are based on *F*^2^, conventional *R*-factors *R* are based on *F*, with *F* set to zero for negative *F*^2^. The threshold expression of *F*^2^ > σ(*F*^2^) is used only for calculating *R*-factors(gt) *etc*. and is not relevant to the choice of reflections for refinement. *R*-factors based on *F*^2^ are statistically about twice as large as those based on *F*, and *R*- factors based on ALL data will be even larger.

### Fractional atomic coordinates and isotropic or equivalent isotropic displacement parameters (Å^2^)

**Table:**

	*x*	*y*	*z*	*U*_iso_*/*U*_eq_	Occ. (<1)
Zn1	0.0000	1.0000	0.0000	0.03753 (14)	
N1	−0.1335 (3)	1.0823 (3)	0.1368 (2)	0.0516 (6)	
N2	0.1925 (2)	0.9400 (2)	0.11113 (18)	0.0398 (5)	
N3	0.3170 (2)	0.8611 (2)	0.26349 (17)	0.0374 (4)	
N4	0.4133 (3)	0.8369 (3)	0.1727 (2)	0.0561 (7)	
N5	0.2223 (3)	0.2854 (2)	0.3054 (2)	0.0431 (5)	
N8	0.0899 (2)	1.2105 (2)	−0.06854 (18)	0.0397 (5)	
N9	0.0847 (3)	1.4448 (3)	−0.1391 (2)	0.0579 (7)	
N10	0.2207 (3)	1.3860 (2)	−0.17586 (18)	0.0395 (5)	
O1	0.5990 (11)	0.4298 (9)	0.9817 (9)	0.135 (3)	0.50
C2	0.1872 (3)	0.9224 (3)	0.2261 (2)	0.0404 (5)	
H2	0.1043	0.9494	0.2736	0.049*	
C3	0.3328 (3)	0.8857 (4)	0.0833 (2)	0.0527 (7)	
H3	0.3701	0.8829	0.0070	0.063*	
C4	0.3613 (4)	0.8227 (3)	0.3821 (2)	0.0444 (6)	
H4B	0.3159	0.8913	0.4273	0.053*	
H4A	0.4742	0.8302	0.3812	0.053*	
C5	0.3110 (3)	0.6738 (2)	0.4421 (2)	0.0343 (5)	
C6	0.3413 (3)	0.6377 (2)	0.5581 (2)	0.0350 (5)	
H6	0.3910	0.7036	0.5933	0.042*	
C7	0.2990 (3)	0.5056 (3)	0.6222 (2)	0.0357 (5)	
C8	0.2262 (3)	0.4067 (3)	0.5681 (2)	0.0396 (5)	
H8	0.1982	0.3171	0.6103	0.048*	
C9	0.1954 (3)	0.4408 (3)	0.4526 (2)	0.0385 (5)	
C10	0.2379 (3)	0.5756 (3)	0.3897 (2)	0.0381 (5)	
H10	0.2168	0.5993	0.3121	0.046*	
C11	0.1173 (3)	0.3323 (3)	0.3945 (3)	0.0466 (6)	
H11B	0.0839	0.2503	0.4527	0.056*	
H11A	0.0251	0.3744	0.3599	0.056*	
C14	0.3404 (3)	0.4707 (3)	0.7471 (2)	0.0458 (6)	
H14A	0.4382	0.4187	0.7498	0.055*	
H14B	0.3564	0.5592	0.7753	0.055*	
C15	0.0108 (4)	1.3348 (3)	−0.0744 (3)	0.0528 (7)	
H15	−0.0874	1.3420	−0.0360	0.063*	
C16	0.2207 (3)	1.2478 (3)	−0.1330 (2)	0.0412 (6)	
H16	0.3017	1.1857	−0.1465	0.049*	
C1	−0.1657 (3)	1.0544 (3)	0.2354 (2)	0.0422 (6)	
N6	0.3491 (3)	0.2042 (3)	0.3333 (2)	0.0560 (6)	
N7	0.3519 (4)	0.2694 (3)	0.1395 (3)	0.0728 (8)	
C12	0.4207 (4)	0.1989 (4)	0.2304 (3)	0.0599 (8)	
H12	0.5134	0.1490	0.2217	0.072*	
C13	0.2266 (5)	0.3210 (4)	0.1909 (3)	0.0661 (9)	
H13	0.1508	0.3756	0.1514	0.079*	
H1A	0.517 (7)	0.391 (8)	1.021 (7)	0.099*	0.50
H1B	0.588 (10)	0.5203 (18)	0.969 (8)	0.099*	0.50
S1	−0.21123 (13)	1.00586 (11)	0.37384 (7)	0.0710 (3)	

### Atomic displacement parameters (Å^2^)

**Table:**

	*U*^11^	*U*^22^	*U*^33^	*U*^12^	*U*^13^	*U*^23^
Zn1	0.0382 (2)	0.0368 (2)	0.0329 (2)	0.00232 (16)	−0.00183 (16)	0.00624 (15)
N1	0.0540 (14)	0.0583 (15)	0.0387 (12)	0.0090 (11)	0.0033 (11)	−0.0010 (11)
N2	0.0371 (11)	0.0417 (11)	0.0377 (11)	0.0018 (9)	−0.0051 (9)	0.0024 (9)
N3	0.0422 (11)	0.0363 (10)	0.0313 (10)	0.0001 (8)	−0.0048 (8)	0.0018 (8)
N4	0.0436 (13)	0.0825 (19)	0.0403 (12)	0.0183 (12)	−0.0039 (10)	−0.0054 (12)
N5	0.0470 (12)	0.0392 (11)	0.0464 (12)	0.0006 (9)	−0.0147 (10)	−0.0112 (9)
N8	0.0418 (11)	0.0370 (11)	0.0364 (10)	0.0007 (9)	−0.0020 (9)	0.0040 (8)
N9	0.0670 (16)	0.0378 (12)	0.0624 (16)	0.0086 (11)	0.0138 (13)	0.0002 (11)
N10	0.0450 (12)	0.0347 (10)	0.0358 (10)	−0.0016 (9)	−0.0034 (9)	0.0023 (8)
O1	0.147 (7)	0.097 (5)	0.142 (7)	−0.010 (5)	0.029 (6)	0.012 (5)
C2	0.0390 (13)	0.0412 (13)	0.0391 (13)	0.0037 (10)	−0.0015 (10)	−0.0019 (10)
C3	0.0417 (15)	0.079 (2)	0.0343 (13)	0.0104 (14)	−0.0010 (11)	−0.0011 (13)
C4	0.0587 (16)	0.0396 (13)	0.0333 (12)	−0.0075 (12)	−0.0109 (11)	0.0017 (10)
C5	0.0352 (12)	0.0323 (11)	0.0340 (11)	0.0014 (9)	−0.0028 (9)	−0.0019 (9)
C6	0.0365 (12)	0.0337 (11)	0.0345 (12)	−0.0018 (9)	−0.0060 (9)	−0.0034 (9)
C7	0.0360 (12)	0.0356 (12)	0.0336 (11)	−0.0013 (9)	−0.0033 (9)	−0.0004 (9)
C8	0.0412 (13)	0.0328 (12)	0.0425 (13)	−0.0024 (10)	−0.0027 (11)	−0.0003 (10)
C9	0.0358 (12)	0.0368 (12)	0.0439 (13)	−0.0017 (10)	−0.0028 (10)	−0.0098 (10)
C10	0.0435 (13)	0.0380 (12)	0.0330 (12)	0.0014 (10)	−0.0068 (10)	−0.0055 (10)
C11	0.0446 (15)	0.0441 (14)	0.0535 (16)	−0.0052 (11)	−0.0081 (12)	−0.0131 (12)
C14	0.0505 (15)	0.0461 (14)	0.0368 (13)	−0.0133 (12)	−0.0084 (11)	0.0072 (11)
C15	0.0564 (17)	0.0432 (15)	0.0531 (16)	0.0054 (12)	0.0138 (13)	−0.0011 (12)
C16	0.0390 (13)	0.0381 (13)	0.0426 (13)	0.0035 (10)	−0.0054 (11)	0.0050 (10)
C1	0.0423 (14)	0.0388 (13)	0.0457 (14)	0.0095 (10)	−0.0019 (11)	−0.0090 (11)
N6	0.0552 (15)	0.0525 (14)	0.0631 (16)	0.0080 (11)	−0.0175 (13)	−0.0127 (12)
N7	0.089 (2)	0.074 (2)	0.0569 (17)	0.0002 (17)	0.0022 (16)	−0.0172 (15)
C12	0.0532 (18)	0.0542 (18)	0.078 (2)	−0.0016 (14)	−0.0063 (16)	−0.0266 (16)
C13	0.086 (2)	0.063 (2)	0.0518 (18)	0.0154 (18)	−0.0215 (17)	−0.0102 (15)
S1	0.0963 (6)	0.0730 (5)	0.0395 (4)	0.0230 (5)	0.0105 (4)	−0.0065 (4)

### Geometric parameters (Å, º)

**Table:**

Zn1—N8	2.146 (2)	C4—H4B	0.9700
Zn1—N8^i^	2.146 (2)	C4—H4A	0.9700
Zn1—N1	2.149 (2)	C5—C10	1.383 (3)
Zn1—N1^i^	2.149 (2)	C5—C6	1.390 (3)
Zn1—N2^i^	2.196 (2)	C6—C7	1.382 (3)
Zn1—N2	2.196 (2)	C6—H6	0.9300
N1—C1	1.152 (3)	C7—C8	1.398 (3)
N2—C2	1.327 (3)	C7—C14	1.515 (3)
N2—C3	1.344 (3)	C8—C9	1.383 (4)
N3—C2	1.322 (3)	C8—H8	0.9300
N3—N4	1.346 (3)	C9—C10	1.398 (3)
N3—C4	1.455 (3)	C9—C11	1.518 (3)
N4—C3	1.316 (4)	C10—H10	0.9300
N5—C13	1.324 (4)	C11—H11B	0.9700
N5—N6	1.357 (3)	C11—H11A	0.9700
N5—C11	1.452 (4)	C14—N10^iii^	1.459 (3)
N8—C16	1.316 (3)	C14—H14A	0.9700
N8—C15	1.353 (4)	C14—H14B	0.9700
N9—C15	1.314 (4)	C15—H15	0.9300
N9—N10	1.361 (3)	C16—H16	0.9300
N10—C16	1.322 (3)	C1—S1	1.625 (3)
N10—C14^ii^	1.459 (3)	N6—C12	1.317 (4)
O1—H1A	0.853 (13)	N7—C13	1.322 (5)
O1—H1B	0.853 (13)	N7—C12	1.334 (5)
C2—H2	0.9300	C12—H12	0.9300
C3—H3	0.9300	C13—H13	0.9300
C4—C5	1.516 (3)		
			
N8—Zn1—N8^i^	180.0	C10—C5—C6	119.5 (2)
N8—Zn1—N1	89.99 (9)	C10—C5—C4	124.7 (2)
N8^i^—Zn1—N1	90.01 (9)	C6—C5—C4	115.9 (2)
N8—Zn1—N1^i^	90.01 (9)	C7—C6—C5	121.1 (2)
N8^i^—Zn1—N1^i^	89.99 (9)	C7—C6—H6	119.5
N1—Zn1—N1^i^	180.0	C5—C6—H6	119.5
N8—Zn1—N2^i^	85.29 (8)	C6—C7—C8	119.0 (2)
N8^i^—Zn1—N2^i^	94.71 (8)	C6—C7—C14	118.5 (2)
N1—Zn1—N2^i^	88.53 (9)	C8—C7—C14	122.4 (2)
N1^i^—Zn1—N2^i^	91.47 (9)	C9—C8—C7	120.6 (2)
N8—Zn1—N2	94.71 (8)	C9—C8—H8	119.7
N8^i^—Zn1—N2	85.29 (8)	C7—C8—H8	119.7
N1—Zn1—N2	91.47 (9)	C8—C9—C10	119.4 (2)
N1^i^—Zn1—N2	88.53 (9)	C8—C9—C11	120.2 (2)
N2^i^—Zn1—N2	180.00 (11)	C10—C9—C11	120.4 (2)
C1—N1—Zn1	140.3 (2)	C5—C10—C9	120.4 (2)
C2—N2—C3	102.6 (2)	C5—C10—H10	119.8
C2—N2—Zn1	127.61 (18)	C9—C10—H10	119.8
C3—N2—Zn1	128.64 (18)	N5—C11—C9	111.5 (2)
C2—N3—N4	109.9 (2)	N5—C11—H11B	109.3
C2—N3—C4	128.9 (2)	C9—C11—H11B	109.3
N4—N3—C4	121.2 (2)	N5—C11—H11A	109.3
C3—N4—N3	102.6 (2)	C9—C11—H11A	109.3
C13—N5—N6	108.7 (3)	H11B—C11—H11A	108.0
C13—N5—C11	129.8 (3)	N10^iii^—C14—C7	113.3 (2)
N6—N5—C11	121.1 (2)	N10^iii^—C14—H14A	108.9
C16—N8—C15	103.1 (2)	C7—C14—H14A	108.9
C16—N8—Zn1	128.82 (18)	N10^iii^—C14—H14B	108.9
C15—N8—Zn1	126.93 (19)	C7—C14—H14B	108.9
C15—N9—N10	102.7 (2)	H14A—C14—H14B	107.7
C16—N10—N9	109.5 (2)	N9—C15—N8	114.2 (3)
C16—N10—C14^ii^	128.6 (2)	N9—C15—H15	122.9
N9—N10—C14^ii^	121.8 (2)	N8—C15—H15	122.9
H1A—O1—H1B	109 (3)	N8—C16—N10	110.5 (2)
N2—C2—N3	110.2 (2)	N8—C16—H16	124.8
N2—C2—H2	124.9	N10—C16—H16	124.8
N3—C2—H2	124.9	N1—C1—S1	176.9 (3)
N4—C3—N2	114.6 (2)	C12—N6—N5	102.2 (3)
N4—C3—H3	122.7	C13—N7—C12	101.6 (3)
N2—C3—H3	122.7	N6—C12—N7	115.9 (3)
N3—C4—C5	114.5 (2)	N6—C12—H12	122.1
N3—C4—H4B	108.6	N7—C12—H12	122.1
C5—C4—H4B	108.6	N5—C13—N7	111.6 (3)
N3—C4—H4A	108.6	N5—C13—H13	124.2
C5—C4—H4A	108.6	N7—C13—H13	124.2
H4B—C4—H4A	107.6		

Symmetry codes: (i) −*x*, −*y*+2, −*z*; (ii) *x*, *y*+1, *z*−1; (iii) *x*, *y*−1, *z*+1.
